# Adolescents’ perspectives regarding their communication with reproductive health service providers in Rwanda: an explorative study

**DOI:** 10.1186/s12913-023-10526-3

**Published:** 2024-01-16

**Authors:** Josephine Uzayisenga, Augustin Nshimiyimana, Gerard Kaberuka, Marie Laetitia Ishimwe Bazakare, Valens Mbarushimana, Madeleine Mukeshimana, Aimable Musafili, Laetitia Nyirazinyoye

**Affiliations:** 1 School of Public Health, College of Medicine and Health Sciences, University of Rwanda, P.O Box 3286, Kigali, Rwanda; 2 Department of Psychiatry and Behavioural Sciences, School of Medicine and Pharmacy, College of Medicine and Health Sciences, University of Rwanda, P.O Box 3286, Kigali, Rwanda; 3 Centre for International Reproductive Health Trainings (CIRHT), Michigan University, P.O Box 3286, Kigali, Rwanda; 4 School of Nursing and Midwifery, College of Medicine and Health Sciences, University of Rwanda, P.O Box 3286, Kigali, Rwanda; 5 Department of Paediatrics and Child Health, School of Medicine and Pharmacy, College of Medicine and Health Sciences, University of Rwanda, P.O Box 3286, Kigali, Rwanda

**Keywords:** Adolescents, Communication, Sexual and Reproductive Health, Health Care Providers, Rwanda, Qualitative

## Abstract

**Background:**

Adolescents in low-middle-income countries often face limited access to health information and services due to several different factors. Ineffective communication between healthcare providers and adolescents is among them. This study aims to assess adolescents’ perspectives regarding their communication with reproductive health service providers in Rwanda.

**Methods:**

A phenomenological exploratory qualitative study was used. Eleven focus group discussions were conducted among adolescents aged 10 to 19 years between December 2020 and January 2021. All participants were identified through their respective health care providers in youth-friendly centres available in the Kigali district representing the urban area and Kamonyi district representing the rural area. All interviews were transcribed and translated into English and analysed by using thematic content analysis.

**Results:**

Poor communication between healthcare providers and adolescents was identified and attributed to the judgmental attitudes of some healthcare providers, while good communication was cited by many adolescents as an important key of access to services. All adolescents were eager to access reproductive health services and be educated about reproductive health issues.

**Conclusion:**

Effective communication is essential when it comes to providing reproductive health services, as this establishes a strong relationship between a service provider and an adolescent who wants to talk about their concerns, while poor communication prevents adolescents from asking questions about unknown topics.

## Background

Worldwide, Adolescents suffer from a disproportionate share of reproductive health problems

When adolescents around the world enter puberty, taboos, discomfort, and fear prevent parents and other trusted adults from teaching and communicating relevant information to help adolescents navigate the complexities of their emerging sexuality [1].

In different African regions like East Africa, and Sub-Saharan countries, health Service Providers are reported as not being experienced in adolescent counselling and communication, facing a dilemma, and not being comfortable with providing sexual reproductive health services to adolescents [2]. They are also reported being undecided between their personal feelings, cultural norms, and values and respecting adolescent’s right to access SRH services [3].

In Rwandan culture, adults focus on abstinence-only, and messages given on contraceptives and sexual reproductive health are seen as taboo [4]. Busy work hours leave little room for sexual reproductive health discussions between healthcare providers, parents, and adolescents and this influences adolescents to rely on peer influence and social media as the most common sources of SRH information. Unfortunately, studies have shown that information obtained from these sources is either incorrect, incomplete, or false [4].

Healthcare providers ‘communication skills with adolescents play a vital role in the early detection of issues, provision of emotional support, effective illness management, and health education [5].

Seven per cent of adolescent women aged 15–19 are already mothers or pregnant with their first child [6], this is one of several reasons that lead to the focus on adolescent reproductive health.

Communication of sexual issues such as unintended pregnancies, HIV or other sexually transmitted infections (STIs), and unsafe abortion between adolescents and health care providers is one of the strategies that could encourage adolescents to delay sexual debut or avoid unprotected sexual intercourse [7]. However, different factors hinder healthcare providers’ and adolescents’ communication about sexual matters [8].

One of the studies done in Rwanda [9], identified the gap in the inaccessibility of information and services among Rwandan adolescents. It showed that young people have inaccurate knowledge about SRH and further reported that they experience difficulties in accessing services.

Lack of communication among parents –adolescents and healthcare providers was attributed to less experience in discussions of sexual topics with adolescents, together with the sensitive nature of the topic [9].

There is very little information on sexual communication between adolescents and healthcare providers. While data are available on the basic sexual and public health outcomes (such as the age of sexual debut, number of sexual partners, and condom use), few studies have been published on Rwandan young people’s thoughts, perceptions, and experiences with sexual communication between adolescents and healthcare providers.

This study was to explore the perceptions of adolescents on reproductive health service providers’ attitudes about sexual reproductive health communication, to determine perceived barriers and challenges that hinder communication between adolescents and providers about reproductive health services, this was a phenomenological exploratory qualitative study that used focus group discussions to identify perceptions of adolescents towards their communication with healthcare providers in urban and rural area of Rwanda. The findings will assist policymakers in addressing this dilemma.

## Methods

### Study design

A phenomenological exploratory qualitative study was used. Eleven focus group discussions were conducted among adolescents aged 10 to 19 years between December 2020 and January 2021. All participants were identified through their respective health care providers in youth-friendly centres available in the Kigali district representing the urban area and Kamonyi district representing the rural area. All interviews were transcribed and translated into English and analysed by using thematic content analysis.

### Study site

The study took place in Rwanda in the districts of Nyarugenge and Kamonyi, which represent urban and rural youth centers, respectively.

### Data collection

Adolescents aged 10 to 19, both male and female, were included in the focus group discussions, with the aim of generalizing the results to all young people, regardless of age. The 10–14 age range was included, as some studies have found that adolescents in that age range experience sexual challenges, such as unwanted pregnancies. The adolescents were interviewed about their experiences and challenges regarding communication with healthcare providers when accessing adolescent reproductive health services. One hundred and thirty-two adolescents participated, and the interviews were transcribed and translated into English for analysis.

### Data analysis

A thematic content analysis approach was used to analyze the data. The interviews were read several times to understand the contexts and implications, and meaning units were extracted from the transcripts. These meaning units were condensed and assigned codes and grouped into categories. Three levels of coding were used in this study: Level 1 involved creating codes from the language used by the participants, Level 2 involved clustering the coded data and creating categories, and Level 3 involved deriving a central theme from the categories.

All focus group discussions were held in youth-friendly centres and were conducted in Kinyarwanda, the language spoken by all participants. Written informed consent and assent were obtained from parents/legal guardians and the adolescents themselves. The Principal Investigator cross-referenced Kinyarwanda and English transcripts to ensure consistency in meaning units, codes, and categories.

## Results

Background characteristics of participant’s adolescents shown in Table 1 are for 132 male and female adolescents that participated in 11 focus group discussions ranging in size of 12 participants in each group. Adolescents were aged between 10 and 19 years with the majority being in the range of 15–19 years. A good number of participants 61 out of 132 means 46% had a primary level educational background; 36 with lower high school level; 28 with upper high school level then 7 with no formal education. Regarding participants’ religion, most of the participants were Christians 85.6% and 14.4% were Muslims. As for marital status, a large number of participants (88%) were single.

**Table 1:** Background Characteristics of participants (adolescents)

7Characteristics	Name of youth centres Total number(n)			Total number(n)
	Kamonyi YC (n)	Kimisagara YC (n)	Rafiki youth centre (n)	TN
**Educational background**				
None	0	5	2	7
Primary	18	31	12	61
Lower high school	13	8	15	36
Upper high school	5	4	19	28
**Religion**				
Christian	33	43	37	113
Muslim	3	5	11	19
**Occupation**				
Unemployed	10	34	4	48
Student	21	10	40	71
Housekeeper	2	4	0	6
Hairdresser	1	0	2	3
Technician	1	0	1	2
Electrician	1	0	1	2
**Marital status**				
Single	30	38	48	116
Married	0	6	0	6
Cohabitating	5	2	0	7
Divorced	1	2	0	3
Widower	0	0	0	0
**Age group (years)**				
10–14	12	10	21	43
15–19	24	38	27	89
**Gender**				
Male	17	2	22	41
Female	19	46	26	91

We identified several important themes related to adolescent SRH communication with healthcare providers, which are detailed below. The first theme measured adolescents understanding about reproductive health and identified insufficient knowledge about sexual reproductive health in rural areas while in urban areas adolescents are familiar with reproductive health issues. The second theme is about source and access to reproductive health information which identified peers as the main source of information as the busy hours of healthcare providers and parents do not allow sexual reproductive health discussion between them and adolescents. The third theme was about the attitudes of reproductive health providers and identified the judgemental attitudes of some who are biased by their internal feelings. The fourth theme regarded perceived barriers that hinder communication between adolescents and providers about reproductive health services and those were, Overloaded healthcare providers, the Rwandan culture of taking sex like taboo, the judgmental attitude of many healthcare providers, and the inappropriate setting where the conversation took place have all been cited as barriers that hamper communication between adolescents and healthcare providers. The last and fifth theme was about challenges of sexual reproductive health communication between adolescents and providers this included health care providers who are working without any guidance and adolescents far from youth centres who arrive late and find the services closed.

Therefore, below is a description of each theme,

### Adolescents ‘understanding of reproductive health topic

Based on different responses about sexual reproductive health, most adolescents from rural youth centres have shown less understanding about reproductive health definition compared to their peers attending urban youth centres. The following quotes are from adolescents in rural youth centres of Kamonyi district trying to answer the question regarding reproductive health:

“………………. Uhhhhhhhh, I don't know much about reproductive health. I just know it's when a boy can get a girl pregnant, or when a girl goes through menstruation or has unprotected sex” (a 17-year-old adolescent boy from a rural youth centre).

*“I think it’s the way our bodies work like someone does sports for a girl or a boy in their twenties.”* (a 15-year-old adolescent girl from a rural youth centre).

* “So I think it is like giving birth to more children, starting a physical transformation maybe someone is starting to gain weight, or how to take care of our bodies (*16 years old adolescent girl of rural youth centre).”

* “Thank you I think it’s like when a ten-year-old girl starts to grow up and needs someone to talk to her about family* (a 15-year-old adolescent girl from a rural youth centre).”

The following quotes were gathered from adolescents in urban youth centres and showed that they had a better understanding of the above definition by WHO:

* “………………. I think that reproductive health is when a person starts to feel changes in his body such as changing the voice for boys, menstruation for girls, developing pimples on their faces, and feeling in you that you have grown up (FGD of boys in urban youth centre).”*

* “What I think about reproductive health, is giving an example to girls and boys as they start talking about sex and start talking about how they use a condom, things like that.”* (17-year-old adolescent boy).

* “For me, reproductive health is just like that for a boy or girl, especially a girl when she starts menstruating and she starts to have pain in her abdomen and she starts to have changes in her body” (*18-year-old adolescent girl*).*

* “I think that it is the life of a young girl or boy when they start showing signs of adulthood, in adolescence it means that it is time to get pregnant and it is characterized by changing the voice, or by showing that he is capable, feeling empowered, for a girl the volume of her breasts increases* “(19-year-old adolescent girl).

After their poor knowledge regarding the WHO's definition of reproductive health, adolescents from rural areas were also more likely to have difficulty understanding reproductive health services compared to their peers in urban youth centres.

### Source and access to reproductive health information

Accessibility to information and services by Rwandan adolescents can improve their sexual life. Giving real information about reproductive health to young people would increase their knowledge and prevent them from getting involved in sexual intercourse without thinking about it. The most important place to get real information is health facilities, where health professionals trained for delivering the deepest information on sexual reproductive health are available. However, most of the respondents, who were asked where they got information on reproductive health, had never cited healthcare providers as their source of the information. They mentioned their peers as the main source of information, instead. In addition, some adolescents have reported that the lack of access to information regarding reproductive health was due to overworked healthcare providers, who were busy with other clients. The latter included young adults or adults who were also seeking services related to reproductive health and rights.

*“The information I have on reproductive health was received from my mother when she explained to me how I should behave during the first menstrual period since menstruation could unexpectedly happen” (FGD of urban girl).*

* “At school, they talked about reproductive health but you could find that they mainly focused on girls because they are the ones who have experienced various menstruation challenges, especially for those who had their first menstrual periods “FGD of a rural boy).*

* “I went to a health centre seeking help for symptoms like malaria and had the opportunity to ask a nurse who treated me why my menstruation was so painful. I asked if it was true that having sex might reduce facial acne for girls. He provided me with adequate explanations about all questions I had asked but I had never planned to go to the youth centre and ask the same questions “(FGD of a rural girl).*

### Attitudes of reproductive health providers

A good attitude is crucial when sharing information with young people, especially since that requires you to behave like a young to talk to them and not judge them, but rather help them understand what they don't understand.

Many participants have complained about healthcare providers who don't have a proper attitude toward them when they come for some services, saying that they are judgemental and may tell their parents about it.

Some healthcare providers are also reported to not pay close attention to adolescents who are facing particular problems and are especially afraid of discussing their sexual history with them.

*“I once went to ask why I was in pain and when I saw how the nurse who was treating me talked to me I was scared. I immediately got up and walked away without asking him what I wanted to ask. In the evening when I came home I asked my classmate and he explained to me a little bit” (FGD of an urban young boy).*

* “Sometimes the health care providers are friends of our parents so one may fear that talking to them and revealing everything may affect the relationship with our parents; they may tell your parents about your individual sexual life and when your parents are aware they may blame you instead of talking to you friendly” (FGD of rural boy YC).*

* “On my side, our centres are just trying to deliver reproductive health services properly in general but they don't have enough time to listen to every adolescent”*

### Perceived barriers that hinder communication between adolescents and providers about reproductive health services

Good communication is the key to an honest and perfect conversation between a young person and a health care provider. If it does not work out for a variety of reasons this can be followed by negative consequences on the part of the youth because the true information they would get from the health care providers was disturbed by improper communication and adolescents may go to an unsure source of information.

Overloaded healthcare providers, the Rwandan culture of taking sex like taboo, the judgmental attitude of many healthcare providers, and the inappropriate setting where the conversation took place have all been cited as barriers that hamper communication between adolescents and healthcare providers.

*“I think the first thing that can make communication between a healthcare provider and a young person ineffective is the lot of work they have. For us, at this centre, I have seen that two healthcare providers are alternating and he or she has to wait for people to come for test of HIV” (FGD of an urban boy).*

* “Some healthcare providers are judgemental when you go for some advice they treat you like their child and start blaming you for what you did instead of paying attention to what you want from him or her”.*

* “Our culture is still a barrier for many Rwandans, including healthcare providers because they often think that someone who should know about sexual life is someone who is going to get married and find that when they are planning a conversation there is something they don't want to talk about while adolescents have information on everything they can’t imagine” (FGD of an urban girl).*

* “Preparing for the right place is important because sometimes they talk to us in the public room and ask us if we have any problems. Many of us are ashamed to ask a private question in public and they prefer to keep “(FGD of a rural girl).*

### Challenges of sexual reproductive health communication between adolescents and providers (identified concerns and difficulties)

According to Rwandan culture, reproductive health is a topic that is only talked about by adults, which makes young people afraid to ask questions about their health. Efforts to start youth centres in each District started after identifying many adolescent challenges related to sexual health, and many of them were ashamed to shout out about the sexual violence they face. Even if in each Rwandan district there is one youth centre, health care providers are not enough considering the number of adolescents they want to serve. They don’t have any guide (manual)to follow when they’re presenting reproductive health topics to adolescents.

The lack of manual guiding healthcare providers when delivering everyday sessions, not enough educational and training programs available to teach reproductive health so that adolescents come with prerequisites, and the absence of youth counsellors in each village to guide adolescents before they reach youth centres in addition with the long distance between adolescents and youth centres, so adolescents come late and find services closed.

*“It is good that sometimes a health care provider comes to talk to us about reproductive health but it is not organized, because youth challenges in our country are well known and depend on the group of age adolescents have (FGD of urban YC).*

* “Some of us live far from here and you see someone takes a long journey to come here and there you find a lot of people who come to get tested or take condoms and the adolescent is embarrassed to ask in general and when he/she decides to ask a health care provider individually he/she finds that the provider is tired and failed to listen properly. For me, I can say that an insufficient number of healthcare providers is a big challenge “(FGD of a rural girl).*

*“I see young people ashamed and afraid, especially our sisters, who are afraid to say what they think when a boy comes to ask them to have sex"(FGD of an urban young girl).*

* “Some of the health care providers we have are adult people while they’re receiving young people. so, you find that adolescents don’t feel free to discuss their health issues. At least four youth counsellors are needed in each village to connect with young people and meet them regularly.”*

## Discussion

This study examined adolescents’ perceptions of their Communication with reproductive health service providers in Rwanda. The report from this study provides solid evidence to support providers, educators, researchers, community leaders, and policymakers as they work toward bringing about urgently needed changes to improve adolescent’s access to accurate information, education, sexual and reproductive health services, and how best it can be improved to promote adolescent sexual health from their early age. Clearly, findings from this study showed that a warm and loving welcome of adolescents by health care providers is a fundamental key to open conversation and accurate communication between them as has been evidenced by similar studies done [10, 11].

The evidence from this study showed that Rwandan youth centres are mostly frequented by old adolescents in 15–19 years but with a low level of education(primary) educational background and most of them attended the centres after sexual exposure similar to the study done in 2018 [11].

Considering responses from different adolescents about reproductive health definition, those from rural areas have poor knowledge of reproductive health definition contrary to those interviewed in an urban area where they know some details of reproductive health definition and this is similar to other studies [12–14].

The study evidenced that the source of information that the majority of adolescents highlighted were their parents, sisters, brothers, trusted neighbours, and school teachers while healthcare providers were not mentioned as a source of information regarding reproductive health services. These findings are contrary to those identified by the studies done in2017 [7, 8] which showed that parents are not trusted by their adolescents in a way that they can discuss their reproductive health issues.

Most of the participants did not appreciate the attitudes of health care providers from health facilities saying that they do not have welcoming attitudes as they are judgemental and that some of them are friends of their parents so they may not be confident about what they will tell them. This is similar to the study done in 2018 [2], this identified weaknesses of healthcare professionals in delivering reproductive health messages and it is similar to the study done in 2019 [15].

Perceived barriers that hinder communication between adolescents and healthcare providers are the high workload of staff at youth centres and the judgemental attitude of healthcare providers. Some adolescents are not free to talk about their individual life in public, poor attitude of girls who are ashamed to talk about their sexual life because of culture, the age range of health care providers who are adults, and young people are not feeling comfortable with them, similar findings have been highlighted by the study done in 2014, 2018 [16–18].

The study identified different challenges to poor communication between adolescents and healthcare providers. Some adolescents don’t even have basic knowledge of reproductive health details and this may hinder communication during health education. Many of them, especially girls, are not free to talk about issues related to sexual life. Sometimes, they meet healthcare providers who are not comfortable with adolescents. The way gender words are used in Rwanda has been taken for a long as an abomination in our culture and therefore if one uses it in Kinyarwanda it immediately sounds like an insult. These study findings are similar to the ones done in 2016, 2019, 2014 and 2019 [9, 19–23].

These findings raise the voice that adolescents have many challenges regarding sexual reproductive health especially poor communication with reproductive health workers. Consequently, adolescents are confused and found the information from peers and this not efficient.

## Conclusion and recommendations

Many of the participants reported that they felt healthcare providers from health facilities were unwelcoming and judgmental. Some participants even mentioned that certain healthcare providers were friends with their parents, which made them hesitant to share certain information with them.

According to the findings of this study, a warm and caring approach from health care providers is critical to fostering open communication and accuracy with adolescents, as evidenced by similar studies.

The study identified different challenges to poor communication between adolescents and healthcare providers. Some adolescents don’t even have basic knowledge of reproductive health details and this may hinder communication during health education. Many of them, especially girls, are not free to talk about issues related to sexual life.

The way gender words are used in Rwanda has been taken for a long as an abomination in our culture and therefore if one uses it in Kinyarwanda it immediately sounds like an insult.

This study indicated that a warm and caring approach from healthcare providers is critical for open communication and accuracy with adolescents.

Addressing barriers to communication between adolescents and health care providers would facilitate improved access to sexual reproductive health.

These findings are important to promote safe sexual reproductive health and prevent health outcomes among adolescents.

## Data Availability

The datasets generated and/or analysed during the current study are not openly available to preserve the anonymity of the research participants but are accessible from the corresponding author on reasonable demand.

## Abbreviations

HIV: Human Immunodeficiency Virus
STIs: Sexually Transmitted Infections
SRHS: Sexual Reproductive Health Services
FGDs: Focus Group Discussions
CMHS: College of Medicine and Health Sciences
WHO: World Health Organization
NGO: Non-organized Organization
CHW: Community Health Workers
